# ﻿A new species of *Diplostephium* (Asteraceae, Astereae) from the Atacama Desert, Chile

**DOI:** 10.3897/phytokeys.215.89175

**Published:** 2022-12-13

**Authors:** Sergio T. Ibáñez, Mélica Muñoz-Schick, Rosa A. Scherson, Andrés Moreira-Muñoz

**Affiliations:** 1 Instituto de Investigaciones Agropecuarias (INIA), Centro Regional de Investigación Intihuasi, Vicuña, Chile Instituto de Investigaciones Agropecuarias Vicuña Chile; 2 Museo Nacional de Historia Natural, Casilla 787, Santiago, Chile Museo Nacional de Historia Natural Santiago Chile; 3 Laboratorio de Sistemática y Evolución de Plantas, Departamento de Silvicultura y Conservación de la Naturaleza, Universidad de Chile, Santiago 9206, Chile Universidad de Chile Santiago Chile; 4 Instituto de Geografía, Pontificia Universidad Católica de Valparaíso, Avda. Brasil 2241, Valparaíso, Chile Pontificia Universidad Católica de Valparaíso Valparaíso Chile

**Keywords:** Antofagasta, coast, fog oasis, lomas, molecular analysis, Paposo, taxonomy, Análisis molecular, Antofagasta, costa, lomas, oasis de niebla, Paposo, taxonomía

## Abstract

A new species, *Diplostephiumpaposanum* S.T.Ibáñez & Muñoz-Schick, **sp. nov.**, is described for Chile, extending the southern distribution of the genus. Its position within the genus was confirmed by morphological and molecular data, discussed here. The new species was found in a coastal environment, new to the genus, and is geographically far removed from the other Chilean species, which are from the Andes. The formation where it occurs, known as lomas, acts as a biodiversity refuge in hyperarid environments. The presence of *D.paposanum* in this environment contributes to the evidence of a floristic connection between the Atacama Desert and the Neotropical Andes.

## Abstract (es)

Se describe una nueva especie para Chile, *Diplostephiumpaposanum* S.T.Ibáñez & Muñoz-Schick, **sp. nov.**, extendiendo la distribución austral del género. Su posición dentro del género fue confirmada por datos morfológicos y moleculares, discutidos acá. La nueva especie se encontró en un ambiente costero, nuevo para el género, y está geográficamente alejada de las otras especies chilenas, que son de los Andes. La formación donde habita, conocida como lomas, actúa como refugio de la biodiversidad en ambientes hiperáridos. La presencia de *D.paposanum* en este ambiente contribuye a la evidencia de una conexión florística entre el Desierto de Atacama y los Andes Neotropicales.

## ﻿Introduction

The genus *Diplostephium* Kunth, in the broad sense, is a diverse group of 111 species (Vargas 2011) distributed continuously on high altitudes of neotropical Andes, from Chile to Venezuela, with some isolated species occurring in Costa Rica and Sierra Nevada (Blake 1922; Cuatrecasas 1969; Vargas 2011). The country with the highest number of species is Colombia, with 63 species described (Vargas 2011). Although it is found in many ecosystems, species of this genus prefer highland conditions, such as open shrublands, grasslands, or upper montane forests (Weddell 1855; Cuatrecasas 1969; Vargas and Madriñán 2012; Vargas 2018).

*Diplostephium* was defined by Kunth (von Humboldt and Bonpland 1820), and separated from similar genera by vegetative and floral characters. This diagnosis was complemented by several authors as more species were described. For instance, Weddell (1855) improved the description of some morphological characters, mainly floral, and indicated the tropical Andes as the only habitat of the genus, expressing a level of uncertainty by adding a question mark to the statement. Weddell (1855) also separated the genus based on the synflorescence structure and leaf shape to help the identification, a criterion followed by Blake (1922, 1928) for his classification. The largest contribution to the genus was made by José Cuatrecasas, who described more than 90 species, 65 of them currently accepted (Vargas 2011). In his classification, Cuatrecasas (1969) used the series established by Blake (1922, 1928) and described new series, which were used by Vargas (2011) in his checklist of the genus.

Vargas et al. (2017) found that *Diplostephium* is not monophyletic, reporting two separate clades that are structured geographically. Those species present between latitudes 11°N and 3°S (60 species) were included in the genus *Linochilus* Bentham (Saldivia et al. 2019), whereas the species present between latitudes 1°N and 22°S (48 species) maintained the genus name. Species included in *Linochilus* can be differentiated from *Diplostephiumsensu stricto* (*s. s.*) by the combination of characters as habit, branching pattern, number of capitula, and the length of corolla ray florets, although no character can be used on its own to distinguish between genera (Vargas 2018).

The last molecular study of the genus *Diplostephium* (Vargas et al. 2017) corroborated previous findings (Vargas and Madriñán 2012) that indicated the lack of monophyly of the genus, and certain incongruences between phylogenies obtained with the nuclear versus chloroplast regions. This was evident in the study by Vargas et al. (2017), who used genomic technology and produced sequences for an extensive nuclear region and the whole chloroplast of most of the known species of *Diplostephium*.

Until now, three species of *Diplostephiums. s.* have been recorded in Chile; *D.cinereum* Cuatrec., *D.tacorense* Hieron. and *D.meyenii* Wedd. (Vargas 2011). The last species has been collected at latitude 22°S (Termas de Puritama, Jul 1969, *O. Zöellner 3014* [PUCV]), the southernmost record of the genus observed by us (Moreira-Muñoz et al. 2016). This study reports a new species of *Diplostephiums. s.* found more than 200 km south of the southern limit of the genus distribution (latitude 24°30'S), herein including a newly associated habitat for the genus, which are commonly referred to as lomas. Such formations are located within the Atacama (Chile) and Sechura (Peru) deserts, and in which vegetation depends on coastal humidity for survival (Rundel et al. 1991, 2007; Cereceda et al. 2008). These formations are adapted to satisfy hydric requirements through humidity in the fog contributed by stratocumulus coming from the Pacific Ocean at low elevations (below 1,000 m), vernacularly called *camanchaca*. The effect of the stratocumulus is especially notorious in the southern Antofagasta Region, around the locality of Paposo, where vegetation thrives in a similar way as it does in the lomas formations in Perú (Johnston 1929; Ricardi 1957; Rundel et al. 1991).

## ﻿Materials and methods

### ﻿Fieldwork

**Figure 1.**
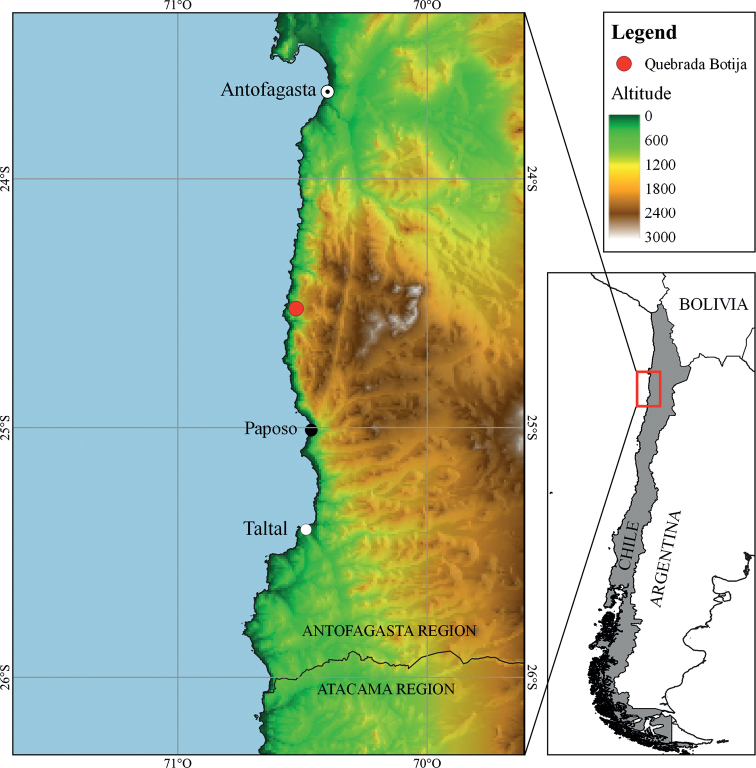
Location of *Diplostephiumpaposanum*. The red dot indicates the location of “Quebrada Botija”, the type locality of the new species.

During a field campaign carried out in December 2020 on the coast of the Atacama Desert, this species was found and collected in Quebrada Botija, an area located in northern Paposo, at an altitude of 170 m (Fig. 1). Afterwards, the location was continuously visited in order to document the full extent of the population including the number of individuals, details of its natural habitat, and to collect seeds for *ex situ* conservation. Photographs in habitat were taken using a camera (Nikon D7500) with macro lens (Sigma 105 mm f2.8G).

### ﻿Study of specimens

Local floras, regional checklists (e.g. Johnston 1929, 1930, 1932; Rundel et al. 1996; Luebert et al. 2007) and herbarium specimens from SGO were revised for the coast of the Antofagasta Region, with emphasis on the family Asteraceae. Chilean *Diplostephium* species were studied, as well as species morphologically similar in leaves size and capitula arrangement (e.g. *D.sagastegui* Cuatrec., *D.foliosissimum* S.F. Blake), for which original descriptions and photographs of taxonomical types observed online (https://plants.jstor.org/) were compared with the new species.

For morphological study, herbarium specimens were dissected and the different morphological components of the plant were studied and recorded for the taxonomic description. Dissected parts were measured under the stereoscopic lens with a scale, and also photographed combining the lens and a camera (Canon G16), and measured afterwards using the software ImageJ (Rasband 1997–2008). Collected and studied specimens were deposited at the herbarium of the National Museum of Natural History (SGO) in Chile. Species nomenclature follows Zuloaga et al.(2008).

### ﻿Molecular analysis

Because of the complexities of the phylogeny previously explained, and the fact that it was not the goal of this study to generate a genomic study, we decided to perform a Barcoding analysis using two commonly used markers for the Asteraceae, comparing the sequences for the new species to the vast number of sequences for the group available in GenBank.

Genomic DNA were isolated from silica gel-dried leaves using the DNeasy Plant Mini Kit (Qiagen, Valencia, CA, USA) following the manufacturer’s recommendations. Two regions were amplified: The internal external transcribed spacer (ETS) region of nuclear rDNA, using primers 18S-ETS (Baldwin and Markos 1998) and Ast8 (Markos and Baldwin 2001), and the chloroplast DNA spacer trnL-trnF using primers “c” and “f” (Taberlet et al. 1991). This combination of primers allowed amplification of a section of approximately 800 bp of this chloroplast region.

For all regions, a polymerase chain reaction (PCR) amplification were performed with 12.5 μL GoTaq Colorless Master Mix (Promega), 2.5 μL of each 10 μM primer, 2.5 μL of 1 mg/ml BSA, 2 μL of DNA template, and distilled nuclease free water for a 25 μL reaction. The PCR reaction followed the protocols of Bonifacino and Funk (2012) for ETS and Taberlet et al. (1991) for trnL-trnF. All PCR reactions consisted of 35 cycles.

Products were purified and sequenced using the Applied Biosystems sequencer ABI3500XL at the Pontificia Universidad Católica de Chile sequencing facility, using the primers described previously at a 5 μM concentration. Both forward and reverse strands were sequenced. Electropherograms of the sequenced products were edited and assembled into contigs using the DNA Baser v4 sequence assembly software (Heracle BioSoft SRL, Pitesti, Romania).

Contigs were used to perform an analysis of sequence similarity using the BLASTn (nucleotide) tool implemented in the National Centre for Biotechnology Information website (http://www.ncbi.nlm.nih.gov/). We used the option of highly similar sequences (megablast), which retrieves all sequences available in GenBank that are highly similar (95% or more) to the target sequence.

### ﻿Conservation assessment

With the information collected through fieldwork and analysed using the software QGIS (QGIS Development Team 2020), the Red List assessment of this species was completed following the guidelines of IUCN (2019).

## ﻿Results

### ﻿Taxonomic treatment

#### ﻿ Diplostephium paposanum

Taxon classificationPlantaeAsteralesAsteraceae

S.T.Ibáñez & Muñoz-Schick sp. nov.

4624F0A8-AB9E-5DFA-B952-DDDAFD0DDBDB

urn:lsid:ipni.org:names:77309837-1

Figs 2 , 3 , 4

##### Diagnosis.

*D.paposanum* is distinctive from most species of the genus because of its lack of tomentose or lanate hairs on its vegetative parts, including the adaxial side of the leaves. Additionally, *D.paposanum* has glandular succulent leaves, and short branches with leaves that are glabrate or scarcely puberulous in the apical section of long shoots.

##### Type.

Chile. Región de Antofagasta: Norte de Paposo, Quebrada Botija, 24°30.334'S, 70°32.834'W, alt. 170 m, 14 Oct 2021. A. Moreira-Muñoz 3355 (holotype: SGO). Norte de Paposo, Quebrada Botija, 24°30.334'S, 70°32.834'W, alt. 170 m, 15 Dec 2020. A. Moreira-Muñoz 3204 (paratype: SGO).

##### Description.

Shrub up to 70 cm tall, subglobose, resinous, generally glabrate but puberulous with hairs mixed with stipitated glands in younger twigs, with ramified erect, indeterminate, ascending, long branches, and shorter, rarely ramified, ascending lateral branches of up to 10 cm borning mostly near the apex of long branches, base of long branches naked, covered with leaves scars. Leaves alternate or fasciculate, densely covering the upper part of the branches and decreasing downward, (1-)2–5 (-8) × 0.3–0.8 mm, succulent, lamina strongly revolute, hence clavate to terete, sessile, covered with sunken glands, younger leaves in plantlets laminar, linear-oblong, with 1–2 teeth at each side of the lamina. Capitula solitary or rarely in pairs, terminal in short lateral branches, heterogamous, radiate. Peduncles (1.5-)2.8–3.9 (-5.5) mm long, often with peduncular bracts 1.0–2.3 × 0.3–0.8 mm, similar to leaves but subulate and base swollen. Involucre 4.1–6.1 × 3.0–4.5 mm, cylindrical; phyllaries arranged in 3–4 series, acute to obtuse, margin hyaline, central rib visible on both sides; outer phyllaries 1.3–2.6 × 0.5–0.8 mm, subulate to deltoid; middle phyllaries 2.4–3.8 × 0.6–0.9 mm, subulate to lanceolate, with or without distal purple spot; inner phyllaries 3.7–4.2 × 0.8–1 mm wide, linear-lanceolate with a distal purple spot; receptacle 1.0–1.5 mm diameter, convex, alveolate, epaleate. Ray florets 7–10, pistillate; corolla white, tube 2.3–4.2 × 0.3–0.4 mm, limb 4.2–7.1 mm × 1.6–2.2 mm, elliptic, minutely 3-lobed; style glabrous, 2.8–4.4 × 0.1–0.2 mm, linear, style branches 0.8–1.7 × 0.1–0.2 mm, linear, flat, with a purple marked line on the middle. Pappus composed of 17–36 bristles of two lengths, short bristles 1.2–2.1 mm, long bristles 3–7.3 mm, scabrid becoming barbellate towards apex. Cypselae of ray florets 1.4–2.9 × 0.4–0.8 mm, shape as in disk florets; carpopodium 1.4–1.9 × 0.4–0.8 mm. Disk florets 10–20, bisexual; corolla yellow, tube 4.3–6.0 × (0.5-)1.0–1.2 mm, narrowly infundibuliform, linear at the base, limb of 5 lobules, each 0.6–1.1 × 0.3–0.6 mm, deltoid; anthers 2.4–3.5 × 0.2–0.3 mm, ecaudate, cuneate, apical appendage deltoid, filaments 1.2–1.8 × 0.05–0.1 mm; style 6.3–7.1 × 0.2 mm, linear, style branches 1.7–2.3 × 0.2 mm, lanceolate, apex acute, distal end straight. Pappus as in disk florets. Cypselae of disk florets 1.7–3.4 × 0.4–0.8 mm, fusiform, slightly compressed, ribbed, villous; carpopodium present, 0.1 × 0.2 mm.

**Figure 2.**
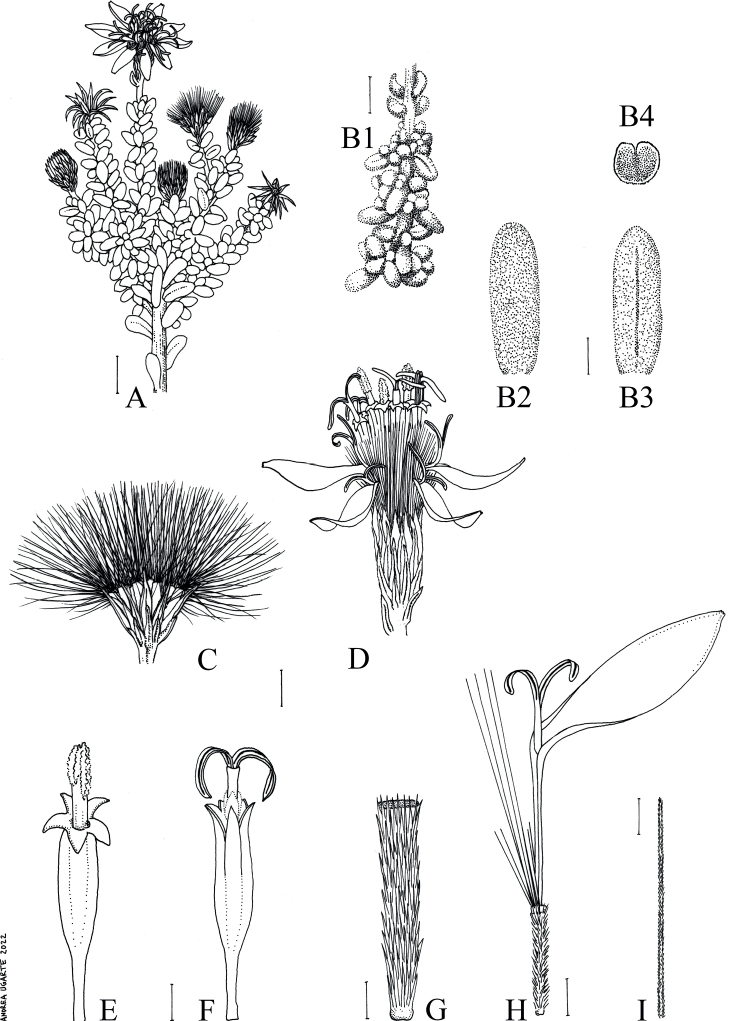
Illustration of *Diplostephiumpaposanum***A** distal end of the branch with capitula **B** details of leaves **B1** upper part of the branch covered by leaves **B2** adaxial view **B3** abaxial view **B4** transversal view **C** capitulum in dispersion of fruits **D** capitulum in bloom **E** disk florets with emerging style branches **F** disk florets with emerged and open style branches **G** detail of a cypsela of a ray floret **H** ray floret, including cypsela. Note the differences in length of pappus bristles **I** detail of a single pappus bristle. All images drawn from *Moreira-Muñoz 3355*. Illustration by Andrea Ugarte. Scale bars: 1 cm (**A**); 5 mm (**B1**); 1 mm (**B2–B4, C–F, H, I**); 0.5 mm (**G**).

**Figure 3.**
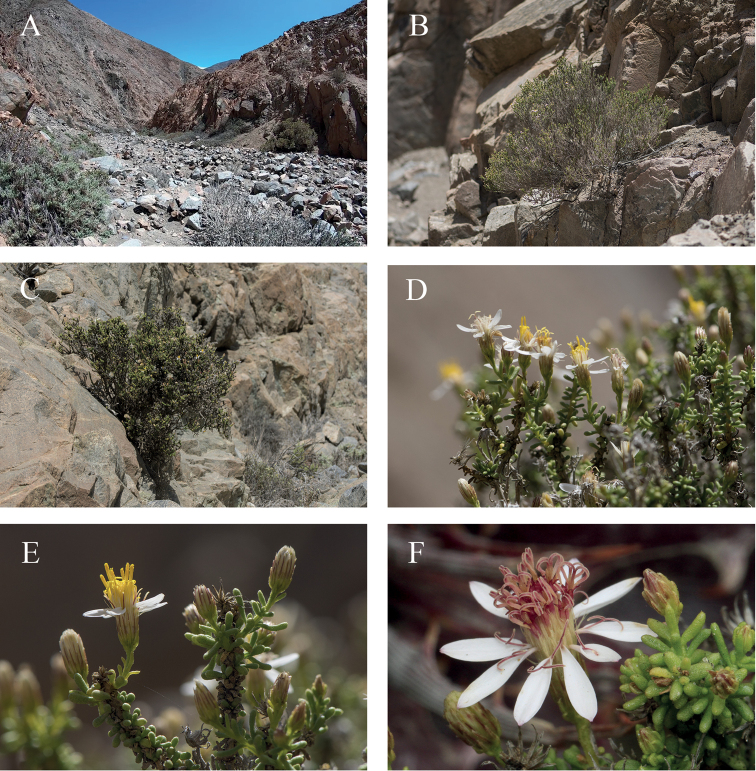
*Diplostephiumpaposanum* in habitat **A** general view of the type location “Quebrada Botija” **B, C** habit of individuals, growing on rocky ledges rooting on rock crevices **D** inflorescences on distal branches **E** lateral branchlets with terminal inflorescences **F** capitulum with fully unfolded style branches **E, F** note the colour differences on yellow disk florets with closed style branches and the mature purple disk florets with unfolded style branches. Photographs by S. Ibáñez (**A–E**), A. Moreira (**F**).

**Figure 4.**
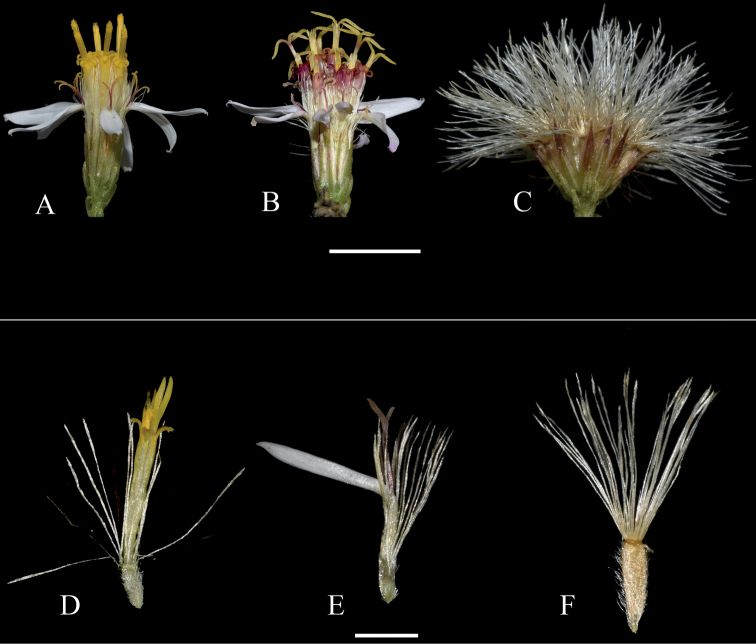
Detail of reproductive organs **A–C** capitula **A** capitulum showing early stages of development, with only anthers of disk florets and style branches of ray florets not unfolded **B** capitulum showing style branches of disk florets unfolded **C** capitulum with mature cypselae and their respective pappus fully developed **D–F** individual florets and fruits **D** disk florets with style branches barely unfolding **E** ray florets with developed style branches **F** mature cypsela of a disk floret. All images from *Ibáñez, Pañitrur & Acosta 771*. Scale bars: 5 mm (**A–C**); 2 mm (**D–F**).

##### Other material examined.

Chile. Región de Antofagasta. Quebrada Botija, 24°31.072'S, 70°31.835'W, alt. 525 m, 4 Dec 2021. S.T. Ibáñez, C. Pañitrur & M. Acosta 771 (SGO).

##### Distribution.

The species has been observed only in Quebrada Botija (24°30'S, 70°32'W), a locality 55 km north of Paposo (Fig. 1), on the coast of the Atacama Desert in the Antofagasta Region of Chile.

##### Habitat.

The only population found was observed in a gorge system, growing from fissures of the ravine walls, or at the bottom of the gorge, often rooting in weathered rocks. The altitudinal range of this species is between alt. 170 and 700 m which suggests that species occurrence is influenced by the presence of the fog from the ocean. It can be found together with species such as *Spergulariaarbuscula* (Gay) I.M. Johnst., *Copiapoaboliviana* (Pfeiff.) F.Ritter, Proustiapungenssubsp.tipia (Phil.) Luebert, *Cristariaintegerrima* Phil., *Eremocharisfruticosa* Phil., and *Jaravatortuosa* (E. Desv.) Peñail. Above the gorges, the vegetation is dominated by *Copiapoasolaris* (F. Ritter) F. Ritter.

##### Phenology.

Flowers of this species have been observed between November and January. Fruits are dispersed between December and February.

##### Conservation status.

The values obtained using the Red List assessment criteria (IUCN 2019) classify this species as “Critically Endangered”, based on the criteria B1ab(iii), C2a(ii) and D. After an extensive search of the area, the only population of this species was found in Quebrada Botija, with an extent of occurrence of 4.6 km^2^ and composed of no more than 20 individuals. Within the area delimited as the extent of occupation, there is a small mining exploitation currently active, in which extracted material is transported using heavy weight trucks moving along the bottom of the gorge in which the species grows. Furthermore, lomas formations are currently threatened by the decreasing amount of humidity reaching the coast in hyper arid Chile, which has led to a notorious declination of vegetation at that latitude (Schulz et al. 2012). Literature and field observation has confirmed vegetation dieback, especially between Botija and Tocopilla (Schulz et al. 2011), where high percentages of individuals of species such as *Copiapoasolaris* are currently dead.

##### Etymology.

The epithet *paposanum* means “from Paposo”. Paposo is a small village located next to the foothills of the coastal cliffs which is the nearest urban centre to the species described.

### ﻿Molecular analysis

After contig assembly and trimming of the primer regions, we obtained 577 basepairs of the ETS sequence, and 824 basepairs of the trnL-F sequence. Both BLAST analyses retrieved highly similar sequences, and both markers obtained species of the genus *Diplostephium* as the most similar. For ETS, the first 40 sequences retrieved were from this genus, with 95% similarity. For trnL-F, sequences retrieved matched species of the genus *Diplostephium* with 98.9% similarity. Within the first 40 sequences retrieved, there were only two that did not correspond to this genus, but were still Asteraceae. The new sequences were uploaded to GenBank, with accession numbers OP038910 for *D.paposanum* trnL-trnF sequence and ON936842 for *D.paposanum*ETS sequence.

## ﻿Discussion

### ﻿Systematic position

The position of *D.paposanum* in *Diplostephiumsensu lato* is supported by morphological characters, such as the shrubby and candelabrum-like habit, heterogamous heads with three to four series of phyllaries and alveolate receptacle, disk florets with ecaudate anthers and lanceolate style branches, ribbed and somewhat compressed cypselae with two different sizes of pappus bristles (Nesom and Robinson 2007; Vargas 2018). Additionally, this species can be placed morphologically in the group of *Diplostephiumsensu stricto* because it is a small bush with up to ten capitula per main branch, all of them placed at the tip of short lateral branches near the apex, and ray flowers often longer than 10 mm. (Vargas 2018). Distribution is also consistent with *Diplostephiums. s.*, since the new species follows the southern distribution of the group, enhancing it southward to latitude 24°S.

Because of the phylogenetic complexity of the genus, we did not infer a phylogenetic position for *D.paposanum*. Nevertheless, we can assume with a high degree of confidence that genetically this new species is most similar to other species of the genus *Diplostephiums.s.* A genomic analysis would be very useful to confirm these results.

### ﻿Biogeographical implications

This species is remarkable because it is the first species of *Diplostephium* collected at low altitude, with an altitudinal range of between 170 and 700 m, whereas all species previously known were collected between 2500 and 4500 m. The new species is similar to some congeneric species, but has distinctive characters which are similar to other coastal species from the Atacama Desert. For instance, *D.paposanum* is a rather glabrate plant with leaves which become succulent and terete to clavate with age. These characters are odd in the genus, but are common in sympatric plants, such as in some shrubby *Nolana* spp. (Solanaceae), *Spergulariaarbuscula* (Gay) I.M. Johnst. (Caryophyllaceae), *Heliotropiumpycnophyllum* Phil. (Heliotropiaceae), or *Suaedafoliosa* Moq. (Chenopodiaceae). The resinous glands present in *D.paposanum* are a common character found in other *Diplostephium* species, which could have led this genus to colonize dry habitats, as has been observed in other xerophytic Asteraceae species.

Despite the fact that it is not common to find high altitude Andean taxa in coastal conditions, some exceptions can be found in the particular conditions of the coastal Atacama Desert, where the coastal fog allows abundant vegetation. This can be seen in *Dalea* species which, when present in South America, are found mostly in the Andes (Piñeros-U and González 2020) with one of the exceptions being *D.azurea* (Phil.) Reiche, which is narrowly distributed around the Paposo coastal area. Also, several phylogenetic analyses show close relationships of some plant groups in the Atacama Desert to Andean and non-Andean neotropical lineages (Luebert 2011; Ruhm et al. 2022). Moreover, numerous species have disjunct distributions, being present in the Andes at high altitudes as well as around Paposo; for instance, species like *Bidenstriplinervia* Kunth, *Steviaphilippiana* Hieron., *Solanumpaposanum* Phil., *Kramerialappacea* (Dombey) Burdet & B.B. Simpson, or *Euphorbiaamandi* Oudejans (Johnston 1929), share this pattern. *Diplostephiumpaposanum*, is the newest component of a remarkable flora recognized by its level of endemism at the Paposo-Taltal coastal fringe, especially in the Asteraceae family (Luebert et al. 2009), a coastal section also recognized as a micro-hotspot of biodiversity integrating new entomological knowledge (Pizarro-Araya et al. 2021).

## Supplementary Material

XML Treatment for Diplostephium paposanum
